# How Within-Study Comparisons can Inform Research and Evaluation Design for the Third Sector

**DOI:** 10.1007/s11266-022-00464-9

**Published:** 2022-02-25

**Authors:** Kaitlin P. Anderson, Patrick J. Wolf

**Affiliations:** 1 Lehigh University, 111 Research Dr, Bethlehem, PA 18015 USA; 2 Graduate Education Building, University of Arkansas, Room 201, Fayetteville, AR 72701 USA

**Keywords:** Within-study comparison, Internal validity, External validity, Quasi-experimental designs

## Abstract

Within-study comparisons (WSCs) compare quasi-experimental results to an experimental benchmark and assess the extent to which quasi-experiments (QEs) sacrifice internal validity for external validity. WSCs inform decisions about when to use experiments or QEs, as well as methodological decisions (e.g., covariate selection) about how to implement QEs when they are necessary or preferred. We review the methodological literature on WSCs and present the findings from a WSC of a school voucher program as an example of the use of WSCs to inform policy debates involving the third sector. Prior literature and our results suggest that QE evaluations of third-sector institutions can produce low levels of bias, but only if analytic models include key proxy variables for voluntary self-selection into programs.

## Introduction

The third sector represents a diverse set of organizations that operate in the social space beyond the market and the state (Salamon & Anheier, 1992), including nongovernmental organizations, charities, voluntary organizations, and not-for-profit organizations (Cordery & Sinclair, 2013). Rigorous evaluation of third-sector organizations (TSOs) is important for proving their worth and justifying continued funding. Further, rigorous evidence is necessary to inform debates about whether services such as education and healthcare should be provided through a single-payer, government-run system, or a public–private voucher system.

Comparisons between the outcomes from government-run institutions with those from TSOs may inform these debates, but selection of participants into various sectors and organizations is a critical consideration. People self-select sectors and organizations based on personal preferences. Eligibility requirements determine who can access services, but even among those who are eligible, service take-up can be hindered by transactional obstacles (Reijnders et al., 2018), or “bureaucratic encounters” (Kahn et al., 1976), with the effect of these barriers distributed unequally across subgroups (Brodkin & Majmundar, 2010). Self-selection is especially relevant to comparisons involving TSOs, as participation is voluntary and many TSOs have distinct identities grounded in social missions or religious traditions attractive to particular clients. Further, some TSOs may carry a social stigma as “charity” (Roberts, 2011), which could influence take-up of services as well.

To address selection issues, randomized controlled trials (RCTs) are the “gold-standard” for estimating the causal impacts of programs and interventions (Mosteller & Boruch, 2002; Rossi et al., 2004). Lottery-based RCTs, if properly implemented, have strong internal validity (Cook & Campbell, 1979). Since everyone in such a study wants to receive service (i.e., “treatment”) from the provider, but only a random subsample of applicants is treated, researchers can be confident that any outcome differences between the groups are due to treatment and not some other factor. Since RCTs are sometimes unethical or impractical, they are typically conducted in special circumstances with distinct subpopulations of participants, affecting external validity, the ability to generalize a specific research finding to a broader population. For example, programs that use lottery-based placement must have demand that exceeds the spots available, so the results may not generalize more broadly (e.g., Abdulkadiroglu et al., 2009).

When RCTs are not possible, or when researchers and policymakers seek to expand the set of participants or programs about which conclusions can be drawn, quasi-experimental (QE) approaches are an attractive alternative. Although typically less internally valid than RCTs, QEs can approximate experimental impact estimates under certain assumptions. Whether these assumptions are fully met is often unknown in practice, and the ability of QE approaches to reproduce experimental estimates is not guaranteed.

Within-study comparisons (WSCs), first introduced by Lalonde (1986), are one useful way to assess the performance of nonexperimental approaches. In a WSC, both experimental and nonexperimental methods are used to evaluate the same intervention, and the nonexperimental methodologies are evaluated for their ability to replicate the experimental estimates. In other words, WSCs test the extent to which nonexperimental evaluation methodologies truly are “quasi” experimental.

In the next section, we discuss some common lessons from the WSC literature. Then, we discuss a WSC from a private school choice program as a demonstrative case. Finally, we conclude with recommendations and implications for third-sector research.

## Key Findings from Within-Study Comparisons

The growing WSC literature covers a variety of fields including education, job training, health policy, criminology, political science, and international development (Wong & Steiner, 2018). Nevertheless, common lessons have emerged. First, nonexperimental estimates are often less biased when individuals in the comparison groups are geographically closer to the experimental sample (Aiken et al., 1998; Bifulco, 2012; Cook et al., 2008; Heckman et al., 1997, 1998a, 1998b; Jaciw, 2016; Shadish et al., 2008). For example, in education, nonexperimental methods are generally less biased when comparison students are selected from the former schools or current neighborhoods of the treated students (Bifulco, 2012; Witte et al., 2014). However, this is not always the case, as Wong et al.’s (2017) synthesis of twelve WSCs as well as a recent study by Unlu et al. (2021) concluded that these types of local restrictions can actually lead to more biased estimates. Such local restrictions may be particularly problematic when the number of close matches available in the locally restricted matching pool is small in number or when selection is primarily due to individual-level factors (e.g., motivation, family involvement), rather than geographic-level factors (e.g., educational resources and opportunities available in a neighborhood or school district). Unlu et al. (2021) concluded that the bias implications of this type of restriction on the potential matching pool is “still an open question” (p. 586). As such, TSO evaluators must evaluate the specific selection mechanisms likely at play when using QEDs, and researchers should conduct more WSCs in a variety of third-sector contexts to help inform these decisions.

Second, covariate selection is critical for reducing selection bias (Smith & Todd, 2005; Shadish et al., 2008; Betts et al., 2010; Cook & Steiner, 2010; Hallberg et al., 2018). For nonexperimental evaluation results to approach experimental estimates, variables related to baseline measures of outcomes and those that predict self-selection must be included in matching algorithms or as control variables (Glazerman et al., 2003; Cook et al., 2008; Cook & Steiner, 2010; Fortson et al., 2012, 2014, Hallberg et al., 2018). For example, when assessing the achievement impacts of educational programs, propensity score matching (PSM) using student demographic variables but without baseline test scores often fails to approximate experimental estimates (Bifulco, 2012; Shadish et al., 2008; Wilde & Hollister, 2007). Notably, Unlu et al. (2021) acknowledge that there is not a “natural pretest” (p. 574) measure available for all outcomes of interest. While test scores or attendance measures may be available for multiple consecutive years, high school graduation or college attendance are one-time outcomes for which baseline measures are either unavailable or only available as a more loosely related measure (e.g., educational aspirations in high school).

Third, methodological approach matters, although no single method is clearly preferred. Researchers and program evaluators should be most wary when a comparison group is not available, such as in a single interrupted time series (Baicker & Svoronos, 2019). Beyond that, methodological choice is still largely an open question. Some methodologists have concluded that PSM reduces bias better than simply using controls in multivariate regression analysis (Fortson et al., 2012, 2014). The performance of PSM, however, can differ due to covariate choice and the characteristics of the pool of potential matches (Bloom et al., 2005; Pirog et al., 2009; Smith & Todd, 2005). The results of WSCs do not always transfer across fields or types of organizations, or even across educational subjects such as math, reading, and vocabulary (Betts et al., 2010; Steiner et al., 2010). Such inconsistencies concern researchers searching for the best alternative approach when experiments are not possible and indicate a continued need for more WSCs involving a variety of different TSOs.

Overall, the WSC literature is discomforting to those hoping to draw causal inferences from QE approaches. A meta-analysis of WSCs finds that even with a rich set of covariates including baseline outcome measures, QE and experimental estimates often differ by policy-relevant magnitudes (Glazerman et al., 2003). To inform researchers, policymakers, and organizations about the appropriateness of using QE methods to assess TSOs, we recommend WSCs be conducted whenever an experimental evaluation makes it possible. Next, we describe the results from one example WSC, assessing the impact of a private school choice program.

## Findings from Anderson and Wolf (2019): School Choice in the Nation’s Capital

In a technical paper, Anderson and Wolf (2019) compare the performance of PSM, kernel matching, and observational models with controls to a set of benchmark experimental results from an evaluation of the District of Columbia Opportunity Scholarship Program (DC OSP) from 2004 to 2009 (Wolf et al., 2010). The federal school voucher program is a prime case study for assessing QE approaches because self-selection is often assumed to play a key role in voucher application and use. Further, the case is useful for understanding the application of WSCs to TSOs more broadly. Although called “private” schools, the participating entities are nonprofit TSOs more accurately described as “independent sector” schools. Voucher programs create a system in which both the government and TSOs educate students using public resources. Thus, evaluations of voucher programs test the relative effectiveness of TSOs and government-run entities in educating children.

Third-sector organizations, in which participation is voluntary, are theorized to be more effective than government providers in many cases and for a variety of reasons. Some scholars point to the fact that people involved in TSOs tend to share a common set of values and beliefs about an organization’s purpose and operating style, fostering a strong sense of mission (Hudson, 1999 in Sarros et al., 2011). Other commentators claim that TSOs tend to outperform government organizations because they eschew the impersonal bureaucracies notorious in government agencies (Ouchi, 1980). Still other scholars finger the absence of political control over TSOs as a major source of their advantage vis-à-vis government organizations, especially in providing K-12 education (Chubb & Moe, 1990).

The empirical literature on the effectiveness of private school vouchers compared to direct government provision of K-12 education is extensive and varied. A meta-analysis of 21 random-assignment studies estimating the impacts of 11 private school voucher programs on math and reading test scores finds a positive impact, on average, with the effects driven primarily by programs in developing countries (Shakeel et al., 2021). Evidence from individual random-assignment studies indicates positive effects of school vouchers on educational attainment (Angrist et al. 2002; Wolf et al., 2013), and altruism toward charitable organizations (Bettinger and Slonim 2006), but negative or null effects on science and social studies scores (Erickson et al., 2021; Abdulkadiroğlu et al., 2018; Muralidharan & Sundararaman, 2015). A recent meta-analysis of the competitive effects of school choice on student achievement system-wide finds small positive effects (Jabbar et al. 2022), suggesting that competition from provision by TSOs pressures government organizations to improve their service delivery. In developing countries, public systems can have particularly high rates of teacher absenteeism, ineffective systems, and mismanagement or leaking of funding targeted for schools, symptoms of a lack of accountability, especially when they are the sole service providers available to citizens (Mbiti, 2016).

Quasi-experimental evaluations of private school voucher programs report findings that tend to be less positive and more varied than those from experiments. The QE effects of vouchers on achievement outcomes range from large positive effects in North Carolina (Egalite et al., 2020) to null effects in Milwaukee (Witte, 2000) and Cleveland (Metcalf et al., 2003) to negative effects in the states of Indiana (Waddington & Berends, 2018) and Ohio (Figlio & Karbownik, 2016). Since none of these evaluations employed the same QE approach, and their results vary among themselves and with the findings from the experimental evaluations, it is important to assess whether the QE evaluations of private school vouchers tend to lack internal validity or whether the experimental evaluations of vouchers tend to lack external validity. The best instrument for doing so is a WSC.

### Methodological Approach

Our WSC of the DC OSP compares experimental results to the results from eight alternative research designs: four kernel matching approaches which differ by estimates and variables used for exact matching, two PSM approaches which differ by variables used for exact matching, and multivariate regression using two different sets of control variables.

The experimental or causal benchmark is the instrumental variable (IV) estimate of the impact of private schooling on math and reading test scores. Lotteries resulted in treatment and control groups with similar baseline conditions (Wolf et al., 2006), but because some control group students “crossed over” to private schooling without a voucher, we use lottery assignment as an instrument to recover unbiased effect estimates of attending a private school (Howell & Peterson, 2006; Murray, 2006). The original OSP evaluators controlled for a variety of background characteristics that were collected as part of the evaluation including baseline test scores, current and prior year grade level, age, household income, number of months at current residence (as a measure of stability), number of children in the household, number of days between September 1^st^ and the date of testing, and indicator variables for gender, Black, mother’s education, mother’s employment status, special education status, and ever having attended a school in need of improvement (Wolf et al., 2010).

Our WSC assesses whether nonexperimental methods would lead to substantively different conclusions about program impacts than this experimental method, and how this divergence depends on the sample used. Specifically, it assesses various methods within two samples:

1. A limited sample of eligible applicants for the program: the experimental or “restricted” sample.
2. A broader sample including treatment units from the experimental sample as well as DC Public Schools (DCPS) students not involved with the RCT as comparison units: the nonexperimental or “unrestricted” sample.

The data available for these two samples differ somewhat. One baseline year and four outcome years are available for the experimental sample, which includes two cohorts of participants in the original study. Only two years of DCPS data (one baseline, one outcome) are available due to a change in the district’s testing administration in 2005–2006. The outcome measures are Stanford Achievement Test—version 9 (SAT-9) scores in both reading and math, standardized by grade and subject. Further, the DCPS data are administrative data collected by the school district for operational and internal reporting purposes and are therefore more limited in scope than the researcher-collected variables in the original OSP evaluation. Ideally, we would have the full set of original control variables used in the OSP evaluation, for use in the WSC as well, but, given the limited nature of the administrative data, we conduct the WSC analysis using the variables available in the administrative data and construct similar variables from the OSP when needed (e.g., using researcher-collected household income to build a measure of FRL-eligibility).

We expect the nonexperimental methods to perform better within the restricted sample, in which comparison units are similar in their eligibility for and motivation to apply to the program, reducing selection bias. Foreman et al. (2019) take a similar approach, assessing the performance of matching methods within a sample of students who applied to attend a charter school, when a lottery analysis was infeasible.

The first nonexperimental method is ordinary least squares (OLS) regression with control variables. For OLS to produce unbiased estimates, all factors confounding the relationship between treatment status and outcomes must be measurable and included in the model (Rosenbaum & Rubin, 1984). This assumption is untestable in practice, but more reasonable if controls for pretreatment outcome measures are included. For the analyses including the broader sample of DCPS students as potential comparison units, a more limited set of controls was available: baseline reading and math test scores, free-and reduced-price lunch eligibility, special education status, limited English proficiency, Black, and current and prior year grade indicators. Our WSC tests the performance of OLS with and without baseline test scores as covariates.

We also assess the performance of PSM. First, we create a set of possible exact matches based on a small set of variables. Within each stratum of exact matches, we calculate a propensity score informed by all other available covariates. Various specifications are assessed, including one exact matching only on grade level, and another exact matching on grade level and special education status. We use 1:1 nearest neighbor matching and require a match within a caliper of 0.1. Within the matched sample, we use OLS regression with the controls available in the DCPS data.

As an alternative to PSM, kernel matching has some better bias and variance properties. Kernel matching (Heckman et al., 1997, 1998a, 1998b) uses multiple comparison units to construct a counterfactual outcome, giving larger weight to comparison units that more closely resemble the treatment units. Kernel matching provides a reduced asymptotic mean squared error relative to pairwise matching (Smith & Todd, 2005) and is more likely than PSM to provide valid inferences when bootstrapping standard errors (Abadie & Imbens, 2008), which is important when comparing across dependent samples (Wong & Steiner, 2018). We perform two types of kernel matching, one exact matching on grade level and another exact matching on grade level and special education status.

A key challenge is assessing the performance of the nonexperimental approaches, relative to the experimental benchmark. One simple way is to count the share of cases in which the same substantive conclusion—positive, negative, or null—would be drawn. Some early WSCs used this approach (LaLonde, 1986; Wilde & Hollister, 2007). However, in Anderson and Wolf (2019), we concluded this method provides little opportunity to distinguish the relative performance of various methods. Further, we might incorrectly conclude that two estimates are similar, even if they are quite different in magnitude, merely because they point in the same direction.

Steiner and Wong (2018) recommend combining statistical equivalence and difference tests (Tryon, 2001). To conclude there is statistical equivalence, both tests must indicate correspondence. To determine statistical difference, both tests must indicate noncorrespondence. When only the equivalence test indicates correspondence, which is more likely with high study power, the two methods produce “trivial differences.” When only the difference test indicates correspondence, perhaps due to low study power, the results are considered “indeterminate.”

To test for significant differences, we use a standard *t*-test for dependent samples, bootstrapping to account for the covariance between estimates (Steiner & Wong, 2018). The null hypothesis is that the difference between the estimates from the experimental and nonexperimental methods equals zero, ${H}_{0}:{\beta }_{NE}-{\beta }_{RCT}=0$. The bootstrapping approach essentially calculates the difference between the estimates from the experimental and nonexperimental methods for each of 500 possible subsamples and uses the distribution of these differences to test the null hypothesis.

To test for equivalence—or more precisely, whether the difference is negligible—we test whether the differences between the estimates from the experimental and nonexperimental methods are small enough to reject the null hypothesis of difference solely due to sampling error at a tolerance threshold, ${\delta }_{E}$. We use a tolerance threshold of 0.1 standard deviation (s.d.) as well as a less conservative threshold of 0.2 s.d. A smaller ${\delta }_{E}$ decreases the likelihood of drawing an incorrect conclusion but increases the likelihood of indeterminacy.

### Key Findings from our WSC

The preferred analyses from our WSC use the Steiner and Wong (2018) correspondence approach and bootstrapping. The performance of various measures is assessed within a restricted sample of eligible applicants and an unrestricted sample that uses DCPS students not involved with the DC OSP as comparison units. Four years of outcome data, in two subjects each, are available for the restricted sample, and only two are available for the unrestricted sample. Many comparisons are made across methods, samples, subjects, and years. Full attention to all the results is not practical here, but we highlight the main findings in the remainder of this section.

The combined statistical tests of equivalence and difference result in one of four conclusions for each comparison of an experimental and a nonexperimental result: a *meaningful difference*, a *trivial difference* less than a chosen threshold of 0.1 s.d or 0.2 s.d., *equivalence*, or *indeterminacy*. Table 1 summarizes the results. No comparisons result in a *meaningful difference* or *trivial difference*, so only reports of *equivalence* and *indeterminacies* are reported. By design, the results using a larger threshold for equivalence are more likely to result in equivalence. The 0.1 s.d. threshold is preferred (Steiner & Wong, 2018) but is more likely to result in indeterminacy.

**Table 1:** Percent of comparisons equivalent at 90% confidence level from correspondence tests

				Kernel Matching		Propensity Score Matching		OLS	
Equivalence Threshold	Subject	Num. Outcomes	IV Results	Exact match on Grade and Spec. Ed	Exact Match on Grade Only	Exact Match on Grade and Spec. Ed	Exact Match on Grade Only	With Baseline Scores	Without Baseline Scores
Panel A: Using only the restricted sample of eligible applicants									
0.1 s.d	Math	4	All Null	12.5%	12.5%	0.0%	0.0%	0.0%	0.0%
	Reading	4	3 Pos., 1 Null	0.0%	0.0%	0.0%	0.0%	25.0%	25.0%
	Both	8	3 Pos., 5 Null	6.3%	6.3%	0.0%	0.0%	12.5%	12.5%
0.2 s.d	Math	4	All Null	50.0%	50.0%	50.0%	50.0%	50.0%	50.0%
	Reading	4	3 Pos., 1 Null	62.5%	25.0%	25.0%	25.0%	75.0%	50.0%
	Both	8	3 Pos., 5 Null	56.3%	37.5%	37.5%	37.5%	62.5%	50.0%
Panel B: Using the larger unrestricted sample including nonapplicants from DCPS									
0.1 s.d	Math	1	Null	0.0%	0.0%	0.0%	0.0%	0.0%	0.0%
	Reading	1	Null	0.0%	0.0%	0.0%	0.0%	0.0%	0.0%
	Both	2	Both Null	0.0%	0.0%	0.0%	0.0%	0.0%	0.0%
0.2 s.d	Math	1	Null	0.0%	0.0%	0.0%	0.0%	0.0%	0.0%
	Reading	1	Null	0.0%	0.0%	0.0%	0.0%	0.0%	0.0%
	Both	2	Both Null	0.0%	0.0%	0.0%	0.0%	0.0%	0.0%

Tests recommended by Steiner and Wong (2018). No results indicated a meaningful difference or a trivial difference, so only equivalence is reported, and the remainder are all indeterminant. Number of outcomes differs based on the years available for the experimental data (4 years) and the supplemental, DCPS data (only 1 year). IV results indicate the substantive conclusions that would be drawn from the experimental, instrumental variables results. Kernel matching summarizes across both average treatment effect (ATE) and average treatment on the treated (ATT) estimates

The main takeaway is that *equivalence* is more common in Panel A, representing the restricted sample, than in Panel B, the unrestricted sample including DCPS students who may not have applied. As hypothesized, the nonexperimental estimates more often resemble experimental ones when the sample is limited to students who are similarly eligible for and motivated to participate in the program. While prior research has focused more on the importance of using comparison units from similar geographic locations (Aiken et al., 1998; Cook et al., 2008; Heckman et al., 1997, 1998a, 1998b; Jaciw, 2016; Shadish et al., 2008), our findings suggest that similarities in other ways—motivation to apply and eligibility for the program—are key for approximating causal results. This may not always be the case, however (Unlu et al., 2021; Wong et al., 2017). For example, if a TSO in the medical field were evaluated compared to a government-run clinic based on the proportion of employees of the two providers who were vaccinated against COVID-19, such a study would need to account for variation in the political ideology of employees across the TSO and government clinic, as ideology is a strong source of vaccine hesitance. TSO evaluators and researchers must carefully consider organization- and context-specific selection dynamics when constructing pools of comparison units. Further WSC research testing the performance of QEDs using different sets of comparison units would help inform the design of more credible quasi-experimental TSO evaluations.

Another major finding of our WSC is that OLS performs better when pre-program measures of the outcome—in this case baseline test scores—are controlled for. Similarly, kernel matching performs better when exact matching on both grade and special education, rather than just grade. This finding reinforces prior literature (Betts et al., 2010; Bifulco, 2012; Cook et al., 2008; Fortson et al., 2012, 2014; Glazerman et al., 2003; Shadish et al., 2008; Wilde & Hollister, 2007) that covariate choice matters, specifically the inclusion of baseline outcome measures. Accordingly, evaluations of TSOs without natural baseline measures should use caution when interpreting quasi-experimental results as causal.

Finally, a third takeaway is that simple OLS regression with control variables sometimes performs better than matching. The variables we use matter more than how we use them. This finding reinforces some prior studies (Betts et al., 2010; Bifulco, 2012) but conflicts with others that suggest that PSM performs better than OLS (Fortson et al., 2012, 2014). Such inconsistencies indicate a need for continued research.

Selection bias is central to these findings. In Anderson and Wolf (2019), when QE approaches are used to estimate program effects among only eligible program applicants, the results often suggest significant positive effects in math, while the experimental estimates suggest null effects. When those same methods are used within the broader sample, including program nonapplicants, the results tend to indicate significant negative effects in math. This suggests that students are negatively selective at the time of application but positively selective at the time of voucher take-up. Negative selection at application might occur if parents seek school choice when their child struggles in their current school due to unobservable factors not captured by baseline achievement (Henig, 1994; Stewart & Wolf, 2014). Positive selection at take-up is consistent with prior research on school choice interventions (Campbell et al., 2005; Fleming et al., 2015; Howell, 2004).

A similar pattern might be expected in a variety of TSOs, particularly those in which eligible applicants may have unmet and difficult-to-measure needs, but more advantaged people in those circumstances might be more likely to take the final steps necessary to experience the intervention. For example, many TSOs have eligibility requirements based on low-income or need, thus intentionally generating negative selection at the application stage. However, individuals may face barriers when it comes to actually following through with a program or intervention (Kahn et al., 1976; Reijnders et al., 2018), which would likely result in positive selection. Or, if the processes used to select and follow-up with applications for treatment allow for cream-skimming or cherry-picking of those most likely to benefit, it would create positive selection bias at this stage. This type of cream-skimming or gaming may be the case particularly in services in which there are strong financial incentives to show effectiveness (Koning & Heinrich 2013). Similar WSCs can further explore these dynamics.

## Recommendations and Implications for the Third Sector

Our advice for the leaders of TSOs is simple. Welcome evaluations of your organization’s performance and use lotteries to determine which motivated and eligible clients gain access to its services. Doing so permits evaluators to use rigorous experimental methods to identify the true difference that TSOs make in the lives of their clients. Gather lots of information about program applicants, preferably before they are served by your TSO, to aid evaluators in establishing reliable comparison groups for any analysis of outcomes.

That advice often will be difficult to follow. However confident the leaders of TSOs are that their organization deliver added value to its clients, putting that expectation to the test takes great courage. Leaders at times may be disappointed in the results. Moreover, most TSOs seek to serve every client who is eligible for their services and motivated to receive it, usually on a first-come-first-served basis. There are some TSO settings, such as the healthcare field, where lottery-based admissions to clinics or services would be unethical in many cases. For that reason alone, QE designs likely will continue to be the dominant methodology for evaluating the performance of TSOs.

Our advice to TSO researchers is more complicated. First, conduct experimental evaluations whenever possible. Experiments yield causal results and lay the foundation for WSCs. Second, where experimental evaluations of TSOs exist or are in the works, researchers should plan to conduct WSCs as follow-ups to shed light on how nonexperimental research in the field can be improved. While ethical concerns may prevent randomization in some cases, conducting a WSC once it has been determined that randomization is feasible and ethical likely adds very little, if any, ethical concern. Indeed, as Cook et al. (2010) have suggested, when concerns about the ethics of random assignment are brought up, we also should ponder the ethics of *not* doing randomized experiments and relying on information based on weak evidence. Just as failing to conduct randomized experiments could be considered unethical in this regard, conducting a WSC, when it is feasible, strikes us as the ethical thing to do.

Third, TSO researchers planning or conducting QE evaluations should draw upon the lessons of WSCs to make their evaluations as plausibly causal as possible. Researchers attempting to evaluate the causal effects of TSOs need to consider the contextual relevance of covariate choice, model choice, sampling frame, and the potential type and degree of selection bias at various stages in the process. Most notably, constructing a comparison group that is similar in important ways, such as interest in and eligibility for a program, is important for reducing selection bias. In some cases, evaluators may consider using multiple comparison groups. These could be composed of similar individuals with demonstrated need being served by another, publicly run service, as well as similar individuals with demonstrated need not receiving the intervention (Caló et al., 2021). However, requiring geographic similarity may actually introduce bias if selection bias is due more to individual-level factors than geographic-level factors or if the pool of potential matches is small (Unlu et al., 2021).

Given the complexity and diversity of institutions within the third sector, researchers must closely evaluate the selection mechanisms that are likely at play in their particular context. For example, selection may operate differently in first-come-first-served TSOs and others that select participants based on need or specific eligibility criteria. The factors that generate selective attrition from programs also should be considered.

Evaluators of TSOs are advised to collect baseline data on as many relevant client characteristics as possible, especially baseline outcome measures, when available. Some health, educational, or other life outcomes relevant to TSO evaluation have multiple measures over time, yet some, such as death or high school graduation, are singular events. In cases of the latter, evaluators should have less confidence in the performance of quasi-experimental approaches. More WSCs should explore the performance of alternative approaches when closely related baseline measures may not be available.

Similarly, while some TSOs have simple interventions and clearly defined and validly measurable outcomes and goals, some may have more complex or subjectively defined goals, such as economic or community development, in which case WSCs may still be fairly limited in their ability to describe the circumstances under which nonexperimental approaches might approximate experimental ones. Additional WSCs should explore this further in a variety of TSOs and with a variety of different types of outcomes.

Researchers attempting to conduct WSCs within the third sector may face some unique challenges. One challenge might be obtaining data from another comparison sector, whether governmental or private. Doing so may be politically difficult, since first and second sector organizations tend to view TSOs as rivals. It also might be legally problematic, as robust privacy protections exist regarding access to personal information in such fields as health care and education. Even when data are available from rival organizations, key variables might be measured differently than they are in the TSO. In our example, we were limited in the number of years of DCPS data for which the same testing outcome was available. Further, even if a robust set of measures are collected within an experimental arm, it may not be feasible to obtain similar variables from comparison units outside the scope of the original evaluation (e.g., DCPS students who never applied to the OSP). Conducting a WSC with a mix of administrative data and researcher-collected data may require some data cleaning and cross-walking to have consistent—albeit limited—measures across different arms of the WSC.

The evidence from WSCs can also help inform researchers, policymakers, and other consumers of research about when it may or may not be reasonable to generalize from unique, single-case experimental findings across a broader set of contexts. This body of work is still growing, and it has some important limitations. However, as interest in WSCs continues to grow, and as the methodological literature on how to conduct well-designed WSCs expands (Cook et al., 2008; Steiner & Wong, 2018; Wong & Steiner, 2018), we expect the capacity of researchers to interpret and meaningfully use the lessons from WSCs to expand as well. Those developments will be especially instructive for rigorous evaluation in the voluntary sector, where individuals are able to self-select into and out of available and accessible programs and providers for which they are eligible. As third-sector organizations continue to serve clients who voluntarily seek them out, causal evaluations informed by the lessons of within-study comparisons can better determine if those organizations are serving them well.
